# A multi-responsive water-driven actuator with instant and powerful performance for versatile applications

**DOI:** 10.1038/srep09503

**Published:** 2015-03-31

**Authors:** Jiuke Mu, Chengyi Hou, Bingjie Zhu, Hongzhi Wang, Yaogang Li, Qinghong Zhang

**Affiliations:** 1 State Key Laboratory for Modification of Chemical Fibers and Polymer Materials, College of Materials Science and Engineering, Donghua University, 201620 (People's Republic of China); 2 Engineering Research Center of Advanced Glasses Manufacturing Technology, College of Materials Science and Engineering, Donghua University, 201620 (People's Republic of China)

## Abstract

Mechanical actuators driven by water that respond to multiple stimuli, exhibit fast responses and large deformations, and generate high stress have potential in artificial muscles, motors, and generators. Meeting all these requirements in a single device remains a challenge. We report a graphene monolayer paper that undergoes reversible deformation. Its graphene oxide cells wrinkle and extend in response to water desorption and absorption, respectively. Its fast (~0.3 s), powerful (>100 MPa output stress, 7.5 × 10^5^ N kg^−1^ unit mass force), and controllable actuation can be triggered by moisture, heat, and light. The graphene monolayer paper has potential in artificial muscles, robotic hands, and electromagnetic-free generators.

Mechanical actuators exhibiting reversible shape changes in response to environmental stimuli, such as light, thermal, electrical, or chemical energy, have attracted much attention. This is because of their potential in environment-triggered sensors, artificial muscles, and electromagnetic-free generators^1^,^2^,^3^,^4^,^5^,^6^. Various actuators including liquid crystal elastomers^7^, dielectric elastomers^8^, ionic polymer-metal composites^9^, and carbon nanotube actuators^10^,^11^ have been developed. However, such mechanical actuators have inherent limitations. Their principle actuation mechanisms have been attributed to stimuli-induced chemical reactions, electrically controlled ion/solvent transport, or thermal expansion. Thus, they require complex chemical systems, external power supplies, or distinct temperature changes (tens to hundreds of °C^11^,^12^), which limits their application. They are also often single-triggered, and exhibit slow responses, small-scale movement, and low stress generation. Moisture-triggered actuators have recently been developed, which are independent of chemical, electrical, and other critical triggers^13^,^14^,^15^,^16^. Pentaerythritol ethoxylate-polypyrrole polymer composites^13^ and carbon-based macroscopic materials^14^,^15^,^16^ have been used for actuators. Shape change is achieved by the desorption and absorption of water to disrupt and reform hydrogen bonds, respectively. These actuators are potential industrial mechanical actuators, but would be difficult to apply in practical devices. This is because their actuation depends solely on humidity, which makes them difficult to control. They also suffer from material (soft matter) and structural (bilayer heterogeneity) limitations that restrict actuation performance. Developing high-performance water-driven actuators that convert easily controlled stimuli (heat or light) into mechanical work remains an important challenge.

Herein, we report a graphene monolayer (GM) paper with a gradient reduced graphene oxide/graphene oxide (rGO/GO) structure. The fabrication process is economical, scalable, and environmentally friendly, and is carried out at low temperature. GO exhibits strong water absorption/desorption capability, and rGO is strongly hydrophobic^15^,^17^. Both GO and rGO exhibit good photothermal heating effects^18^,^19^. These properties are exploited to yield GM paper with reversible, fast (~0.3 s), powerful (7.5 × 10^5^ N kg^−1^ force output), and controllable mechanical deformation and recovery, in response to moisture, heat, and light. The response of this water-driven actuator to multiple stimuli allows the artificial muscles and electric generators to be fabricated. The GM paper can be produced on large scales (i.e. many meters), which promotes its practical application^20^,^21^. Fibre-like actuation devices can only be produced on smaller scales (i.e. microns-meters).

## Results

**Figure 1:**
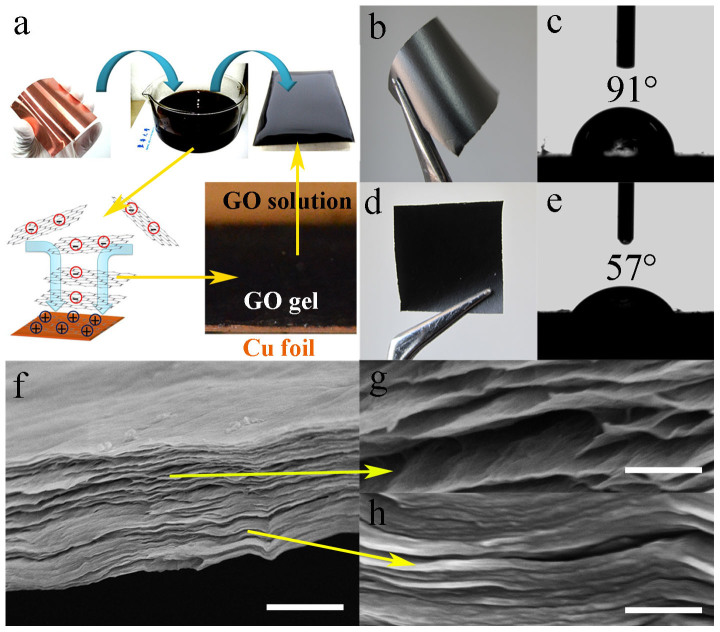
Fabrication and characterization of the GM paper. (a) Schematic of the fabrication process. The Cu foil thickness is 160 μm. (b) and (c) Surface observation and CA measurement of the rGO face of a 17 × 17 mm paper. (d) and (e) Surface observation and CA measurement of the GO face of the paper. (f–h) Cross-sectional FESEM images indicating GO (g) and rGO (h) regions of the paper. Scale bars: 1 μm (f); 200 nm (g and h).

We fabricated GM paper with an asymmetric structure, using a layer-by-layer method modified from previous reports^22^,^23^,^24^. Fig. 1a shows the fabrication of the GM paper. A piece of copper foil was immersed in concentrated GO solution (5 mg mL^−1^, pH = 3). Oxidation and reduction occurred on the copper surface because of the reductive and oxidative nature of copper and GO, respectively (Note S1). More negatively charged GO sheets were then electrostatically attracted from solution to the rGO-covered copper surface. A large amount of copper ions were released from the copper foil during oxidation and reduction^23^. The spontaneous reduction of GO and its assembly were achieved without additional reducing agents. A rGO hydrogel layer assembled on the metal substrate after 24 h at room temperature (Fig. 1a inset). The copper ions neutralized the negative charge of rGO near the copper surface and GO in bulk solution (Fig. S1). The reactions between metallic copper and GO led to the reduction of GO at the bottom of the GO gel, while functional groups remained on the other side (Fig. S2). After subsequent washing and drying, the graphene paper had an asymmetric rGO/GO structure.

**Figure 2:**
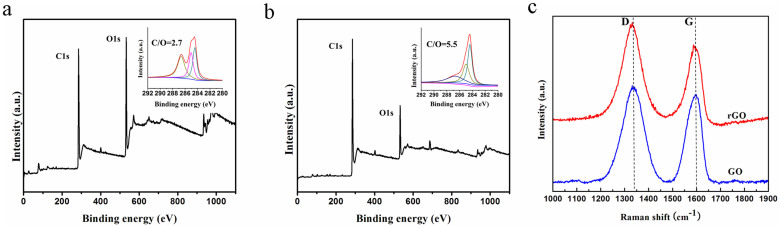
Characterizations of the GM film. (a–b) High-resolution XPS analysis (C 1s) of GO and rGO face of the GM film. c) XRD patterns of GO and rGO surface of the GM film. All the XRD patterns were recorded at room temperature.

Fig. 1b–e show surface observations of the free-standing GM paper. It has one smooth surface with a shiny metallic lustre, while the opposite surface is rough and dark. The static water contact angles (CA) of the two surfaces are about 91 and 57°, indicating hydrophobic rGO and hydrophilic GO structures, respectively. The deoxygenation of GO and rGO faces of the GM paper were proven by the detailed characterization of X-ray photoelectron spectroscopy (XPS). As shown in the XPS spectra (Fig. 2a and b), the C/O atom ratio was remarkably increased from 2.7 of GO face to 5.5 of rGO face. And the deconvoluted C 1s core-level spectra provide details about the surface functional groups present in the GO surface of the GM paper (sp^2^-hybridized graphitic carbons ~284.5 eV, sp^3^-hybridized saturatedcarbons ~285.0 eV, and carboxyl groups ~288.9 eV)^2^. While the C 1s core-level spectra of the face connected with Cu film shows that oxidized species decrease their concentration and the sp^2^ hybridization increases which is typical behavior after reducing GO. Their surface chemistry was also investigated using Raman spectroscopy (see Fig. S2c.). Raman spectra of GO and rGO faces of the GM paper. All spectra show the characteristic D- (1345 cm^−1^) and G-bands (1600 cm^−1^) of those samples. The D-band is quite intense in the rGO face, and the intensity ratios for the D and G bands (*I*_D_/*I*_G_) change from 1.12 (GO) to 1.27 (rGO). The values are both significantly larger than that of original GO sample (0.78)^2^. These results suggested the decrease in the average size for the sp^2^ domains upon the reduction of GO occurred on both sides of the GM paper and a deeper reduction in the rGO face of the GM paper in comparison to its GO face. The reduction of GO is confirmed by the Raman spectra, similar to in our previous works^2^,^20^. The rGO sheets assemble into a compact layered structure, because of the π-π stacking interactions of rGO. This is shown in the cross-sectional field emission scanning electron microscopy (FESEM) images in Fig. 1f and 1h. The rGO region has a high conductivity of 2347 S m^−1^. In contrast, oxygen-containing functionality makes the GO region nearly insulating. The GO region has a cellular structure (Fig. 1g), with sufficient room for large-scale deformation and channels for water exchange.

**Figure 3:**
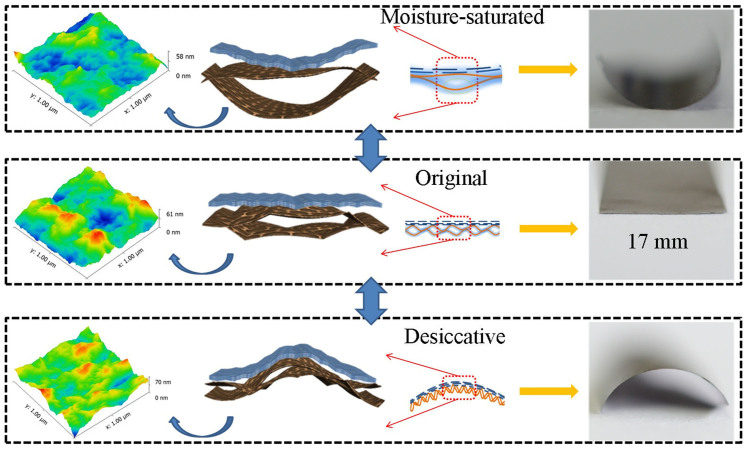
Structural changes in response to water absorption and desorption are shown in the AFM images. The resulting deformation is clearly visible. The blue regions in the sketches indicate the rGO face of the GM paper, while the brown regions indicate the GO face.

The GO region of the GM paper has a cellular structure and oxygen-containing functionality. It can exchange water with the environment, resulting in fast and reversible contraction and expansion. The hydrophobic compacted rGO sheets barely absorb water. The differential expansion/contraction of regions within the paper results in its bending (Fig. 3). Under desiccative conditions (Ar-filled glove box, H_2_O <1 ppm), the removal of water exerts a capillary force on GO, and drives the wrinkling of GO sheets^26^. This leads to contraction of the GO cells, and the GM paper bends toward the GO face. Under humid conditions (relative humidity >80%), water saturates the surface of the GO cells and penetrates the GO layers, which increases the length and thickness of the GO cells^27^. The expansion of the GO face bends the GM paper toward the rGO face. The principal deformation mechanism is proposed to be the water-driven wrinkling/unwrinkling of GO sheets. This is confirmed by the atomic force microscopy (AFM) images in Fig. 3. The three-dimensional topographical AFM image shows regular height fluctuations, which indicate the GO cells (Fig. 3, middle panel). When the GO face of the GM paper is exposed to water vapour, it unwrinkles and exhibits a more straightened surface morphology (Fig. 3, top panel). The maximum height, mean (Ra) roughness, and root mean square (RMS) roughness decrease by 5 (from 61 to 59 nm), 18 (from 7.31 to 5.97 nm), and 20% (from 9.33 to 7.49 nm), respectively. When the sample is dried, it wrinkles and exhibits more irregular creases (Fig. 3, bottom panel). The maximum height, Ra roughness, and RMS roughness increase by 15 (from 61 to 70 nm), 22 (from 7.31 to 8.90 nm), and 19% (from 9.33 to 11.14 nm), respectively. These results and detailed studies on single wrinkles (Fig. S3) indicate the contraction and expansion of GO cells. This process is similar to the deformation of household paper (Fig. S4), but the reversible deformation of the GM paper is faster and more pronounced.

**Figure 4:**
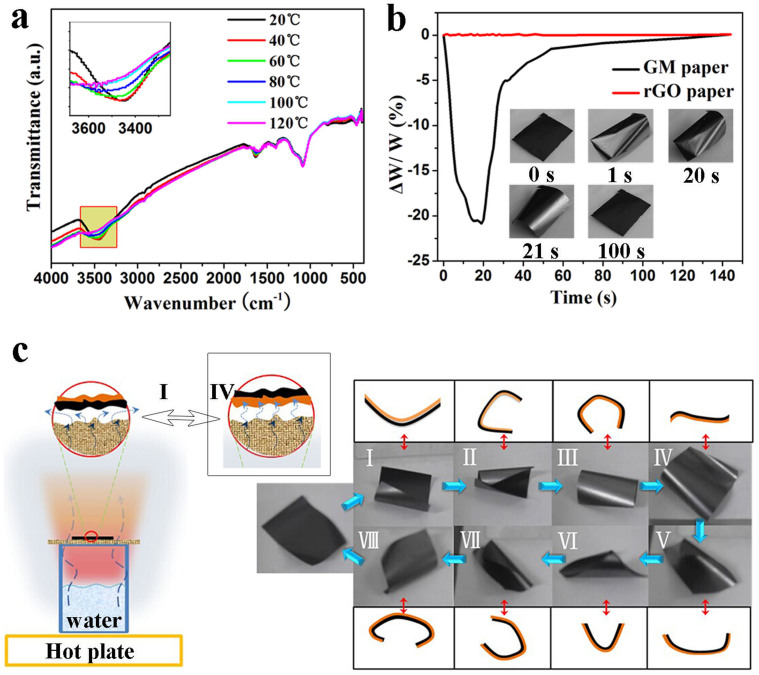
Thermal-responsive behaviour of the GM paper and its locomotion in response to moisture and heat. (a) ATR-IR spectra of the GM paper measured at different temperatures. (b) Time-dependent weight measurements of the paper under on/off heating. Insets show the paper (20 × 30 mm) deformation. It was heated for 20 s and then allowed to cool to room temperature. (c) Representative images and schematics of the paper's multistage locomotion.

Temperature is a more effective trigger than moisture for the current actuator. Attenuated total reflectance-infrared (ATR-IR) spectra (Fig. 4a) of the GM paper show that the intensity of the hydroxyl stretching vibration of water decreases with increasing temperature. Fig. 4b shows the GM paper rapidly loses up to 20% of water by weight, as the temperature increases to 55°C. It gradually absorbs up to 20% water by initial weight, upon cooling to room temperature in humid air. Therefore, we further investigate the water molecule content-dependent bending angle of the actuator. The specific water molecule number change (ΔS*_N_*, Note S2) and the bending angle show near-linear dependency (Fig. S5). Conventional rGO paper exhibits negligible weight changes and deformation during the same test. This suggests that only the GO regions of the rGO/GO structured paper are responsible for actuation. A small change in surface temperature (~4°C from 0 to 1 s; ~1°C from 20 to 21 s) results in significant deformation of the actuator (Fig. 4b inset). This could be attributed to water being easily removed from the GO surface^26^, and free water easily transferring into cellular channels. The asymmetric response of the two faces enables the rGO/GO monolayer paper to exhibit a rapid, well-controlled and continuous motion in a pre-established manner. The GM paper can spontaneously and continuously flip and navigate over a vapour-heated filter membrane, when triggered by both moisture and heat (Movie S1 and Fig. 4c). A typical cycle occurs in two stages: 1) When the rGO region faces down, the GM paper resists water vapour but conducts heat. The GO region is thus heated and contracts, causing the GM paper to curl away from the substrate (Fig. 4c I–III); 2) once the paper flips and the GO region faces down, there exist two deformation mechanisms—the dominant moisture-induced swelling of GO, and the secondary thermal-induced deswelling of GO. The swelling effect is much stronger, so that the paper begins to uncurl (Fig. 4c IV) and then curl in the opposite direction. The paper then flips again, so that the rGO region faces down (Fig. 4c V–VIII), and a new cycle begins.

**Figure 5:**
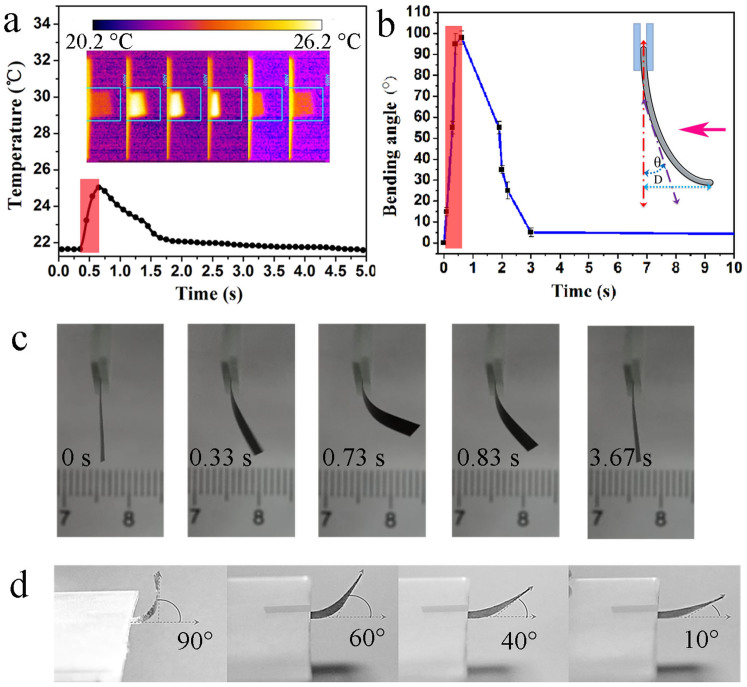
Light-responsive behaviour of the GM paper. (a) Time-dependent surface temperature measurements of the paper under on/off AM 1.5 irradiation, where irradiation occurs for 0.15 s (red area). Insets show corresponding infrared thermal images. The maximum temperatures within the marked 25 × 25 mm area were used. (b) Time-dependent bending angle (indicated by pink arrow) of the paper under on/off AM 1.5 irradiation, where irradiation (1 sun) occurs for 0.5 s (red area). The error bars indicate standard deviations calculated from repeated measurements. (c) Light response of the paper actuator. A visible and near-infrared laser is incident for 0.73 s from the right side of the image. d) Bending angle measurements of the paper under different illumination intensities (from left to right: 150, 100, 50, and 10 mW cm^−2^). Simulated solar light is incident for 0.3 s from the top of the image.

Graphene exhibits photothermal effects^18^,^19^, so the GM paper exhibits fast and reversible photo-induced actuation. Fig. 5a insets show infrared thermal images of the GM paper and its reversible deformation under illumination. Simulated air mass (AM) 1.5 illumination generates a small but observable increase in the maximum surface temperature of the GM paper (ΔT = 4°C, Fig. 5a) within 0.15 s, which is sufficient to bend the paper considerably. The paper reverts to its relaxed state within 3 s after illumination is removed, as its surface temperature decreases. Thus, the shape of the GM paper can be controlled by short-duration on/off illumination. The photoactuation is clearly visible in Fig. 5c and Movie S2,S3 and S4. Conventional graphene papers do not exhibit such ‘smart’ behaviour (Fig. S6).

The bending angles (*θ*) and displacement (D) of the actuator were determined based on the model shown in Fig. 5b inset. The *θ* and D were captured and plotted as a function of time. Fig. 5b shows that *θ* increases with irradiation, and reaches 95° within 0.3 s. The actuation response time constant for this bending is <0.3 s (Note S3), which is faster than light-triggered polymer (10 s)^28^ and carbon nanotube-based bilayer (0.5 s)^11^ actuators. The bending and relaxing of the GM paper are reproducible (Fig. S7), and the paper is stable upon exposure to air and ambient humidity for 18 months. The water exchange capability of GO cells results in the GM paper being highly sensitive to light. Fig. 5d shows *θ* as a function of light intensity. Irradiating the paper for 0.3 s at 10 mW cm^−2^ (0.1 sun) results in a *θ* of 10°. The *θ* begins to saturate at 90°, when the illumination intensity is >150 mW cm^−2^.

**Figure 6:**
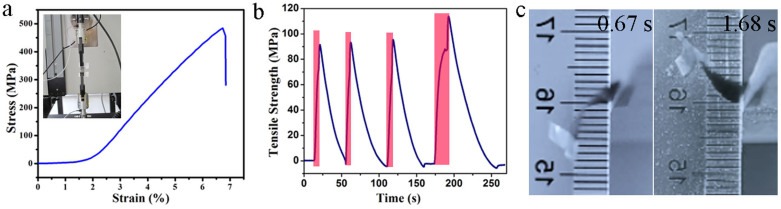
Mechanical performance of the GM paper. (a) Stress-strain curves of the paper actuator. (b) Time-dependent stress generated by irradiating the paper actuator with an infrared laser (red areas). (c) Images of the actuator lifting a metal film by 12 mm within 1.01 s under irradiation. Light is incident from the top of the image.

The strength of the actuator and its generated force and stress were measured on a universal testing machine (Fig. 6a inset and Fig. S8). The actuator was cut into 2 × 20 mm strips, which were clamped and preloaded with a stress of 0.01 MPa, to keep them tight and straight. An infrared laser (Philips BR125) was used to irradiate the actuator. Fig. 6b shows that with prolonged irradiation time, the stress generated by the actuator (with an ultimate tensile strength of 500 MPa) exceeds 100 MPa (4 N) (after deducing the pre-stress). This is almost three orders of magnitude higher than that of mammalian skeletal muscle (0.35 MPa)^13^, and much higher than the maximum stress electrochemically or thermally generated in polymer artificial muscles (~35 MPa)^12^,^29^. After deduction, the output force and unit mass force are as large as 4 N and 7.5 × 10^5^ N kg^−1^ respectively (Fig. S8b), which is significantly higher than those of the azobenzene (a Liquid-crystalline elastomers) (0.14 N)^30^ and an adult's arm muscle (~180 N kg^−1^)^30^. Conventional graphene paper provides zero stress during the test. Upon irradiation, a 0.5-mg GM paper can deform and lift a 13-mg load by 12 mm within 1.01 s (Fig. 6c and Movie S5). The power density is 3.1 W kg^−1^, which is comparable to that of a reported moisture-triggered polymer actuator (2.5 W kg^−1^)^13^.

## Discussion

**Figure 7:**
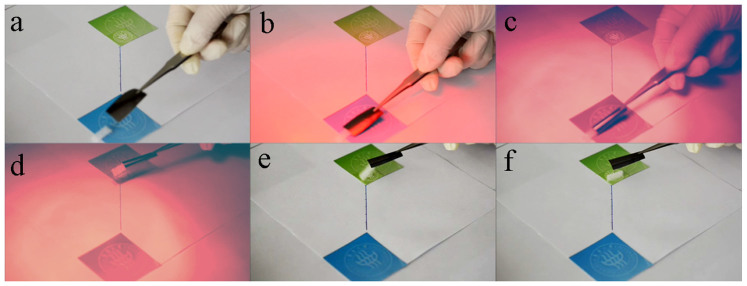
Light-responsive robot hand. (a–f) Optical images showing a GM paper-based gripper driven by infrared light. It curls to grip an object (b), and moves by 100 mm (c, d) under light irradiation. After removing the infrared light, it rapidly opens to drop the object (e, f).

An actuator-based gripper was fabricated to demonstrate the potential of the GM actuator for muscle-like behaviour. Fig. 7a–f and Movie S6 show typical gripping-moving-releasing action. The free end of the actuator could close/open under on/off infrared irradiation, which provides the driving force for gripping/releasing objects, respectively. The performance of this graphene actuator-based artificial muscle (or robotic hand) is repeatable (Movie S7) and flexible (Movie S8).

**Figure 8:**
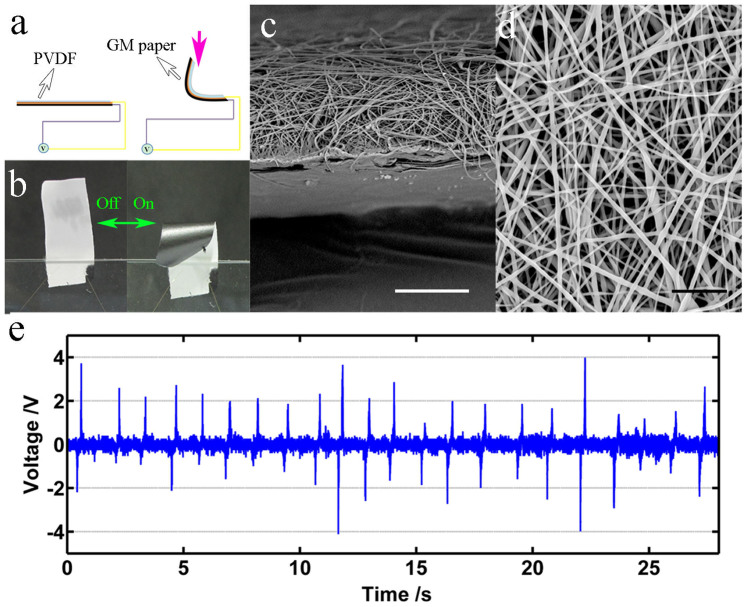
Design and performance of a light-responsive generator. (a) Schematic of the designed generator. Pink arrow indicates irradiation. The electrodes are on both sides of PVDF film. (b) Optical images of the generator (20 × 50 mm) under on/off irradiation. (c) Cross-sectional FESEM image of the hybrid generator. Scale bar is 15 μm. (d) FESEM image of the PVDF face of the generator. Scale bar is 5 μm. (e) Light-responsive voltage curves for the generator.

The GM paper can continuously convert photon energy into mechanical work, and can then drive a piezoelectric element to further convert the mechanical energy into electrical energy. Combining these two energy conversions may provide an alternative means for converting solar energy to electricity compared with current solar cells. A 13-μm-thick piezoelectric poly(vinylidene fluoride) (PVDF) film (Fig. S9) was attached to the GO face of the GM paper, via an electrostatic spinning method. Fig. 8a–e shows a schematic, photographic and FESEM images, and photo-electronic behaviours of the graphene paper generator. The typical bending/unfolding behaviour is observed under on/off AM 1.5 irradiation. The deformation of the device stretches the PVDF element, and generates an open-circuit voltage of up to 4 V, as measured by an oscillometer. The frequency of the alternating voltage is 0.8 Hz, which matches the motion frequency of the generator.

In conclusion, the reported GM paper exhibits fast and powerful actuation performance, in response to moisture, heat, and light. A light-driven robotic hand and generator have been demonstrated as representative applications of the GM paper.

## Methods

### GM paper fabrication

All the reagents were of analytical grade and used as obtained without further purification. The GO was synthesized through the Hummers' method^31^. A piece of copper foil (0.16 mm in thickness) was immersed in a GO solution (5 mg mL^−1^, pH = 3). A rGO hydrogel layer was assembled on the metal substrate after 24 h at room temperature (25°C). It was then repeatedly dried and washed at room temperature. Finally, a free-standing GM paper was peeled off from the copper foil.

### Graphene/PVDF generator fabrication

The hybrid paper-like generator was prepared using an electrospinning technique based on our previous report^25^. In brief, PVDF powders (FR904) were dissolved in a dimethylformamide (DMF)/acetone mixture (DMF/acetone = 6/4 w/w) at the polymer/solvent weight ratio of 1/9. JG50-1 Model HV power supply (Shanghai Shengfa Detection Instrument) was used. The applied voltage was 18 kV. The spinning solution was placed in a hypodermic syringe and was delivered to the blunt needle tip at a flow rate of 1 mL h^−1^ via a micro-syringe pump (KDS101) at a fixed collecting distance (15 cm) between the tip of the syringe and the GM paper collector.

### Characterizations and measurements

The morphologies of the as-prepared samples were determined using a JSM-6700F FESEM (JEOL), and the photographs were taken using a single-lens reflex camera (Nikon D7000). XPS measurements were performed using a Kratos AXIS ULTRA Multifunctional X-ray Photoelectron Spectroscope and the typical detection depth is ~5 nm. All XPS spectra were corrected using the C 1s line at 284.6 eV. Raman spectra were recorded on a Renishaw in plus laser Raman spectrometer with λ_exc_ = 785 nm. The contact angle measurements were conducted using a contact angle meter (Contact Angle System OCA40, Dataphysics Co.) at ambient temperature. 5 μL deionized water was dropped on the samples using an automatically dispense controller, and the contact angles were determined automatically by using the Laplace-Young fitting algorithm. The electrical conductivity of the paper samples were measured using the 4-point probe method (MCP-360, Mitsubishi Chemical Analytech Co. Ltd.). AFM images were taken out using a Nanoscope IV SPM (Digital Instruments). ATR-IR spectra were recorded on a Nicolet NEXUS-670 spectrometer. A Mettler Toledo AL204 laboratory balance was used to collect mass data. The temperatures and infrared thermal images were recorded using an FLIR Thermo-Vision A40M infrared thermometer. The force and stress generated by the graphene actuator as well as its strength were measured on a universal testing machine (Instron Model 5969). Output voltage was measured by an oscillometer (Wavesurfer 104MXs-B, LeCroy). Simulated sunlight was provided by a Newport solar simulator (Model 96160) with an AM 1.5 G filter. An infrared laser (250 W, Philips BR125), and a visible and near-infrared laser (400–1100 nm, 20 W max, SFOLT Co. Ltd.) were also used to irradiate the actuator.

## Author Contributions

C.H. and J.M. contributed equally. C.H. and J.M. performed the experiments. C.H., J.M. and H.W. conceived and designed the experiments. Y.L., B.Z. and Q.Z. analysed the data. C.H., J.M. and H.W. wrote the manuscript. All authors reviewed the manuscript.

## Supplementary Material

Supplementary Information Supplementary information

Supplementary Information Supplementary Movie S1

Supplementary Information Supplementary Movie S2

Supplementary Information Supplementary Movie S3

Supplementary Information Supplementary Movie S4

Supplementary Information Supplementary Movie S5

Supplementary Information Supplementary Movie S6

Supplementary Information Supplementary Movie S7

Supplementary Information Supplementary Movie S8
