# Supplementary material for: A multi-responsive water-driven actuator with instant and powerful performance for versatile applications
Source: Sci Rep. 2015 Mar 31;5:9503. doi: 10.1038/srep09503 (PMC4379955; doi:10.1038/srep09503)
Supplement: Supplementary Information [file srep09503-s1.doc]

Supplementary Information

**A multi-responsive water-driven actuator with instant and powerful performance for versatile applications**

*Jiuke Mu*,^1,†^ *Chengyi Hou*,^1,†^ *Bingjie Zhu*,^1^ *Hongzhi Wang*,^1^,*^*^ Yaogang Li*,^2^ *and Qinghong Zhang*^1^,*^*^*

1 State Key Laboratory for Modification of Chemical Fibers and Polymer Materials, College of Materials Science and Engineering, Donghua University, 201620 (People’s Republic of China)

2 Engineering Research Center of Advanced Glasses Manufacturing Technology, College of Materials Science and Engineering, Donghua University, 201620 (People’s Republic of China)

^†^ These authors contributed equally to this work.

*^*^* Correspondence and requests for materials should be addressed to H. Wang (email: [wanghz@dhu.edu.cn](mailto:wanghz@dhu.edu.cn)) or to Q. Zhang (email: zhangqh@dhu.edu.cn).

**Supplementary Figures**


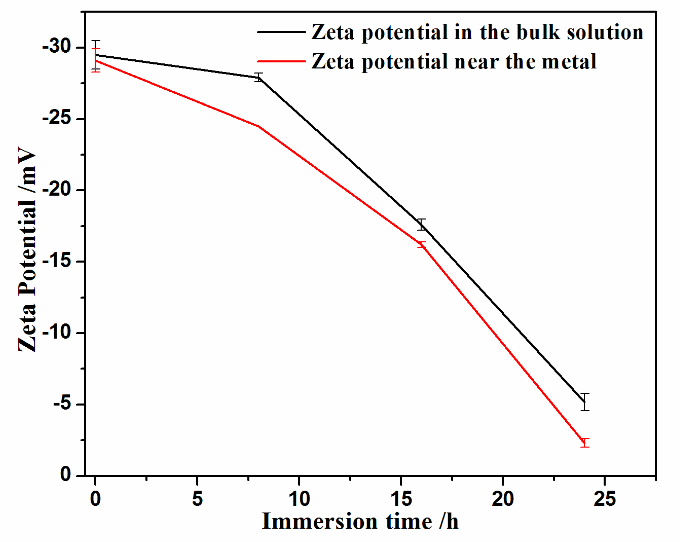


Supplementary Figure S1. Zeta potential as a function of immersion time of the copper substrate. The bulk solution and the solution near the copper were monitored. The error bars are the standard deviations calculated from repeated measurements.


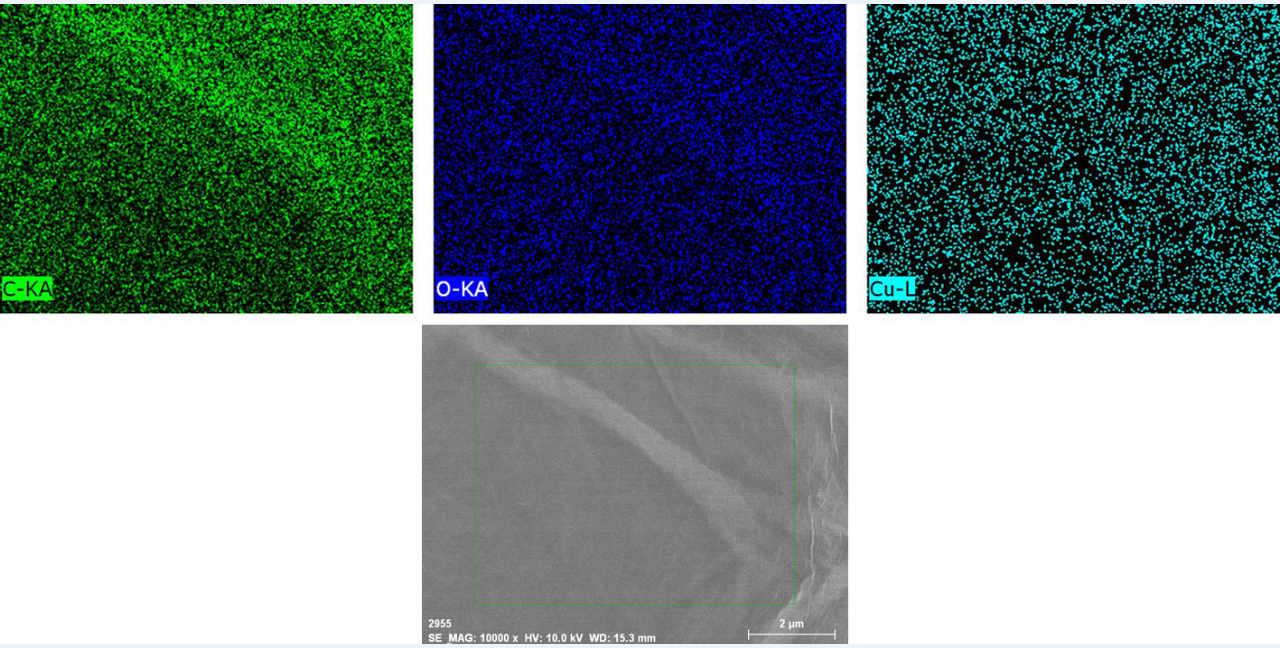


Supplementary Figure S2. Energy dispersive spectrometer (EDS) mapping images of signals from C, O, and Cu element for GO face of the graphene monolayer (GM) paper.


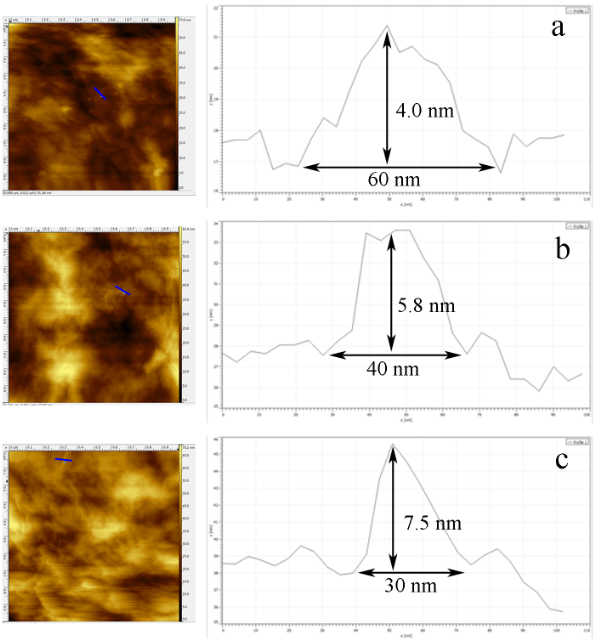


Supplementary Figure S3. AFM images and cross-section profile for the GO face of the GM paper under different conditions: a) moisture-saturated, b) original, and c) desiccative. The size of the single wrinkles estimated from the cross-section profiles is about 60 (a), 40 (b), and 30 nm (c), with a height of 4.0, 5.8, and 7.5 nm, respectively. This strong indicates that water molecules remove from GO surfaces and the GO cells wrinkle themselves (a–c). The process is reversible according to the results shown in the main text.


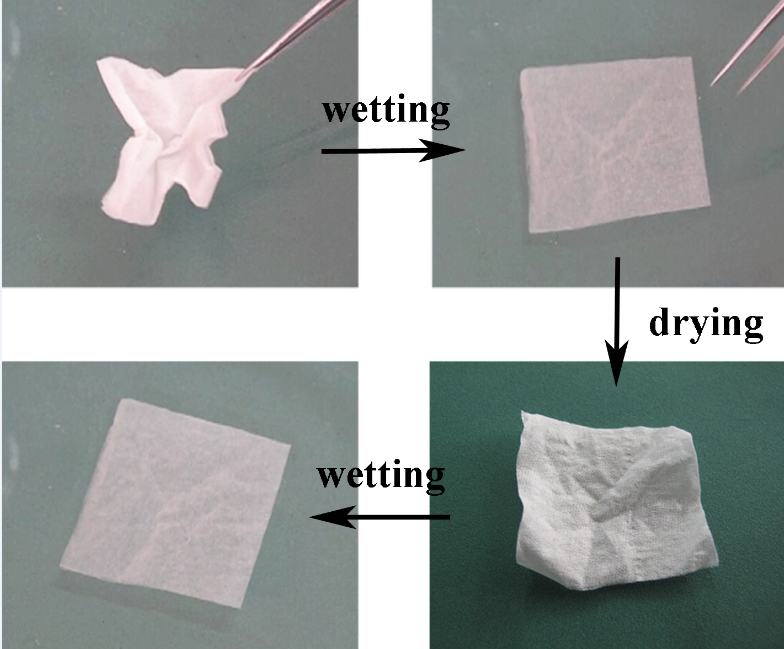


Supplementary Figure S4. When a piece of wrinkled paper is placed on a bath of water, it unwrinkles itself and eventually straightens; and conversely, when a piece of straightened wet paper is dried, it wrinkles itself. This is a common phenomenon in our lives. The principle mechanism is understood as the differential water-driven expansion/contraction of a layered structure of the paper. This is an inspiration to us for designing the GM paper.


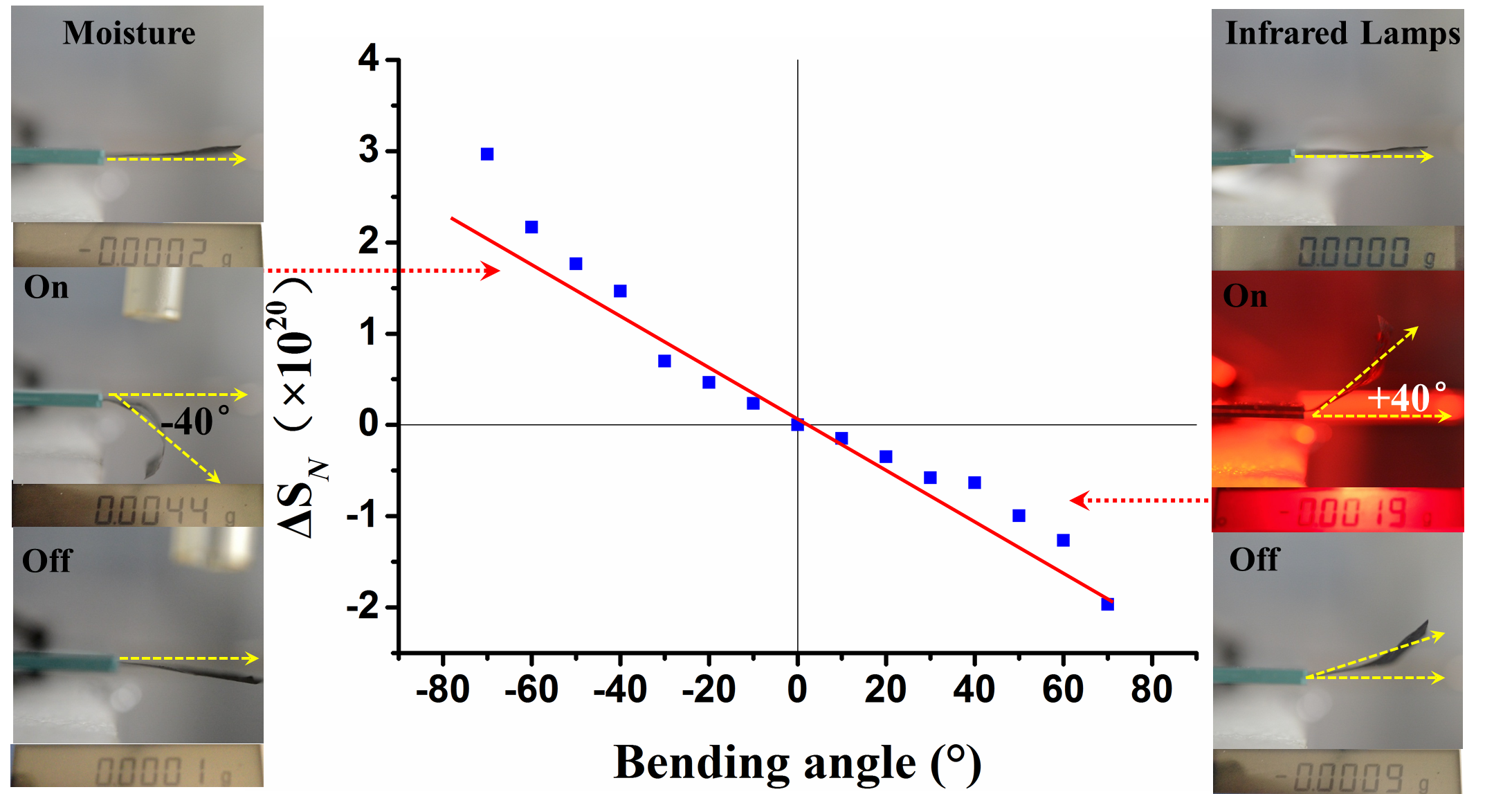


Supplementary Figure S5. The bending angle measured at different specific water molecule number changes (ΔS*_N_*, Note S3) of the GM paper. The bending angle is controlled by the irradiation of infrared lamps (-) and the introduction of moisture (+).


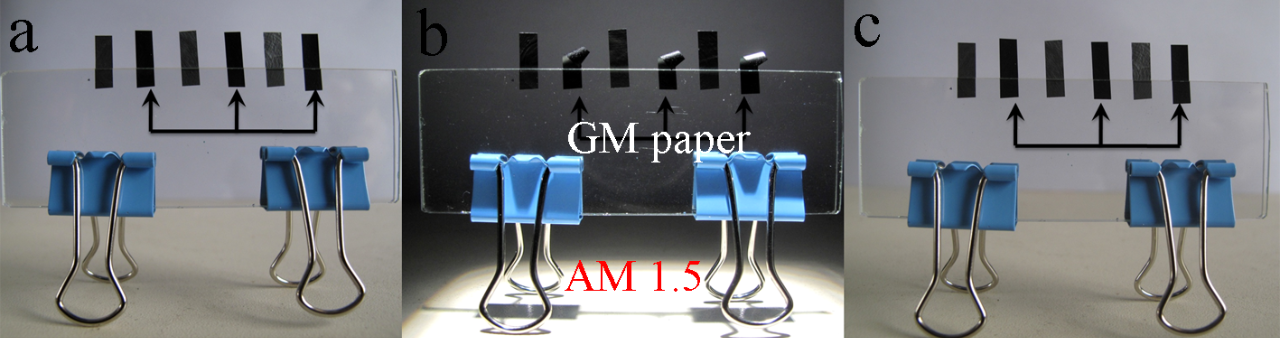


Supplementary Figure S6. Different light-responsive behaviors of GM papers and conventional graphene papers fabricated through previous methods^2^.


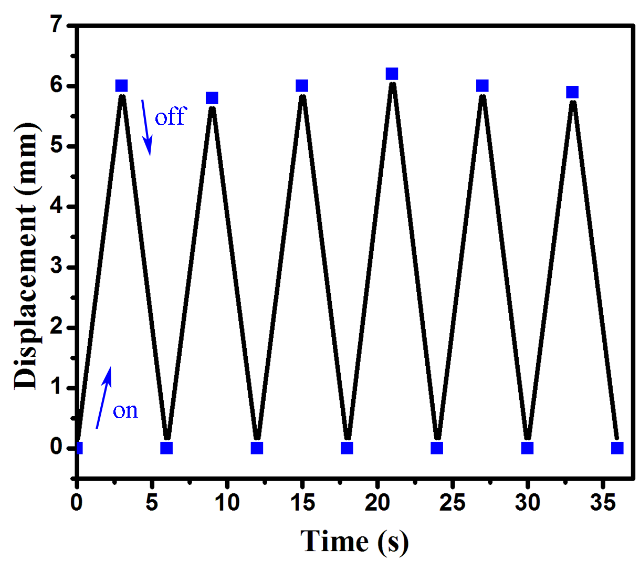


Supplementary Figure S7. The displancement changes of the GM paper in responsive to on/off simulated solar irradiations.


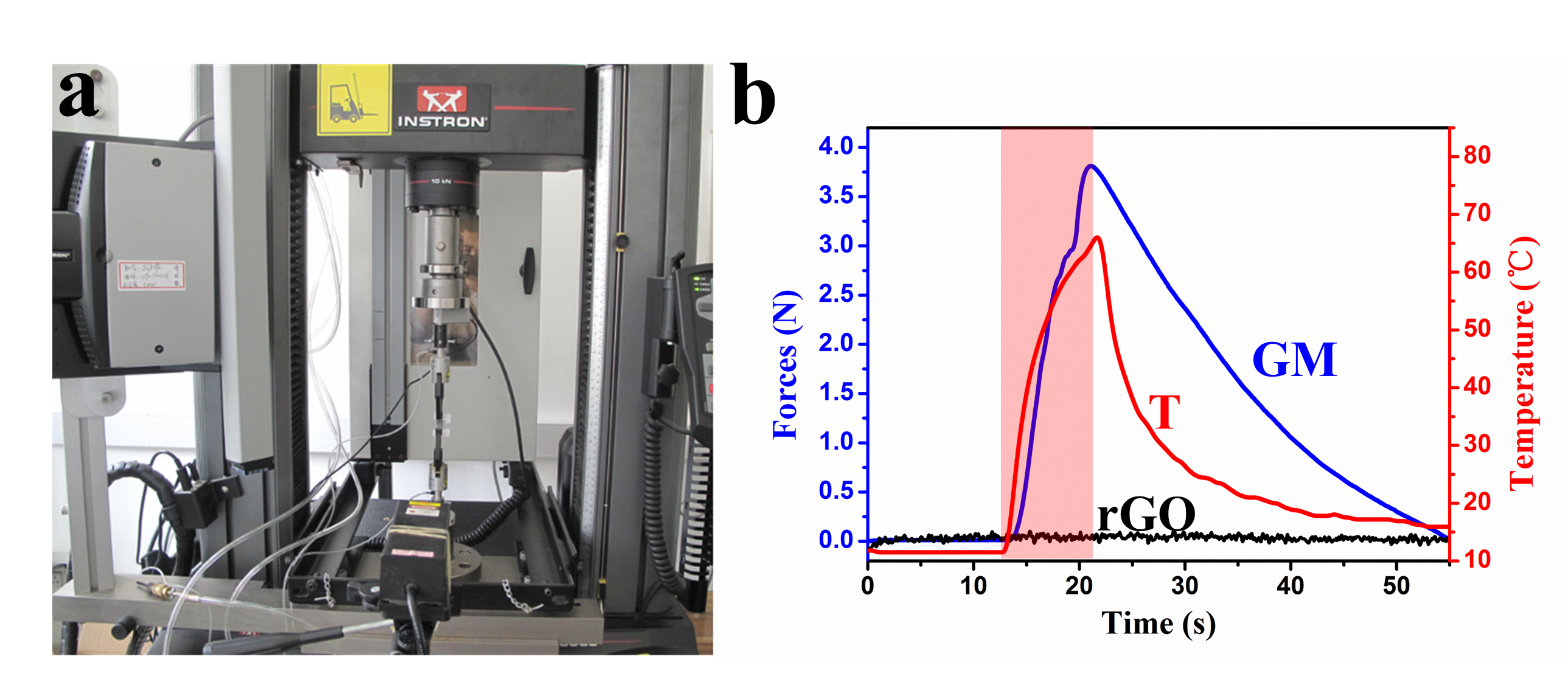


Supplementary Figure S8. a and b) The force generated by the graphene actuator (blue curve) and rGO film (black curve) were measured on the universal testing machine (Instron Model 5969) shown in the figure. The surface temperature (red curve) of the GM paper is measured at the same time.


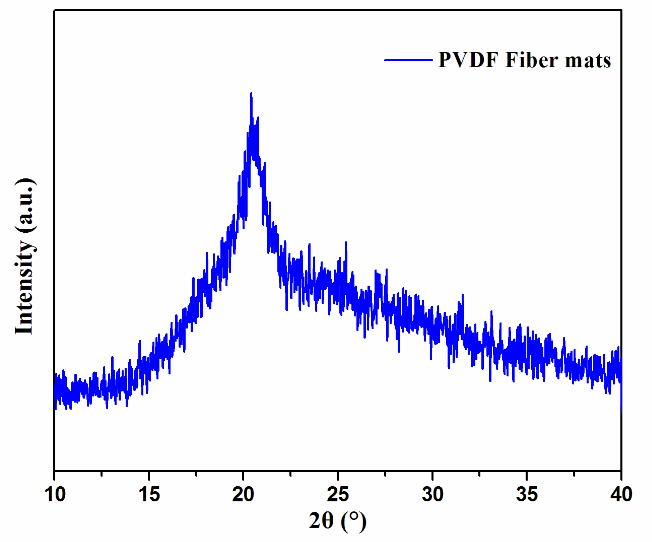


Supplementary Figure S9. X-ray diffraction (XRD) pattern of the as-prepared PVDF nanofiber mat. The XRD result indicates that piezoelectric β-PVDF nanofibers were prepared through the electrospinning technique^3^.

Supplementary Notes

Supplementary Note S1. Oxidation-reduction reactions occurred on the surface of the copper foil.

Once GO encountered copper, oxidation-reduction reactions occurred^4^, as shown below:

Cu+GO+2H^+^=Cu^2+^+rGO+H_2_O (1)

Supplementary Note S2. Calculation of the specific water molecule number changes (ΔS*_N_*) of the GM paper.

The specific water molecule number changes (ΔS*_N_*) of the GM paper can be calculated using expression (2)

ΔS*_N_* = (Δm_GM_/M) * NA (2)

where Δm_GM_ represents the weight change of GM paper, M is the relative molecular weight of water molecules, NA is Avogadro's number.

Supplementary Note S3. Calculation of the actuation response time constant.

The bending process can be fitted with an exponential response using equations (3)^5^:

*θ*=*θ*_0_[1-exp(-*t*/*τ*)] (3)

where *θ* is the bending angle, *θ*_0_ is the maximum bending angle, *t* is time and *τ* is the actuation response time constant.

Supplementary Methods

Zeta potential measurements were performed on a Zeta Potential Analyzer (Nano ZS, Malvern). EDS measurements were performed on a JSM-6700F FESEM (JEOL). Raman spectra were recorded on a Renishaw in plus laser Raman spectrometer with λ_exc_=785 nm. XRD spectroscopy was carried out on a Rigaku D/max 2550 V X-ray diffractometer using Cu K_α_ irradiation (λ=1.5406 Å). The operating voltage and current were kept at 40 kV and 300 mA, respectively.

Supplementary Movies

Supplementary Movie S1. A GM paper is flipping and navigating over a vapor-heated filter membrane.

Supplementary Movie S2. The photoactuation behavior of the GM paper. A relatively high-power visible-near infrared laser radiation (200 mW cm^-2^) is incident from the right side of the scene.

Supplementary Movie S3. The photoactuation behavior of the GM paper. A relatively low-power visible-near infrared laser radiation (20 mW cm^-2^) is incident from the right side of the scene.

Supplementary Movie S4. The repeated photoactuation behavior of the GM paper.

Supplementary Movie S5. Upon AM 1.5 light irradiation, a 0.5 mg GM paper is deforming and lifting a 13 mg load to a height of 12 mm within 1.01 s.

Supplementary Movie S6. Driven by infrared light, a GM paper is gripping and moving an object.

Supplementary Movie S7. Driven by infrared light, a GM paper is repeatedly gripping and moving objects.

Supplementary Movie S8. Driven by infrared light, a GM paper is gripping and moving an object in another way.

Supplementary References

1. Pei, S., Zhao, J., Du, J., Ren, W. & Cheng, H.-M. Direct reduction of graphene oxide films into highly conductive and flexible graphene films by hydrohalic acids. *Carbon* 48, 4466–4474 (2010).
2. Hou, C. *et al.* A strong and stretchable self-healing film with self-activated pressure sensitivity for potential artificial skin applications. *Sci. Rep.* 3, 3138 (2013).
3. Cao, X. *et al.* Ambient fabrication of large-area graphene films via a synchronous reduction and assembly strategy. *Adv. Mater.* 25, 2957–2962 (2013).
4. Zhang, X. *et al.* Photoactuators and motors based on carbon nanotubes with selective chirality distributions. *Nat. Commun.* 5, 2983 (2014).
